# Chrysosplenetin inhibits artemisinin efflux in P-gp-over-expressing Caco-2 cells and reverses P-gp/MDR1 mRNA up-regulated expression induced by artemisinin in mouse small intestine

**DOI:** 10.1080/13880209.2016.1241810

**Published:** 2016-12-08

**Authors:** Liping Ma, Shijie Wei, Bei Yang, Wei Ma, Xiuli Wu, Hongyan Ji, Hong Sui, Jing Chen

**Affiliations:** a School of Pharmacy, Ningxia Medical University, Yinchuan, Ningxia, PR China;; b Institute of Clinical Pharmacology, General Hospital of Ningxia Medical University, Yinchuan, Ningxia, PR China

**Keywords:** Polymethoxylated flavonoids, antimalarial drugs, ABC transporters, P-glycoprotein

## Abstract

**Context:** CYP3A4 and P-gp together form a highly efficient barrier for orally absorbed drugs and always share the same substrates. Our previous work revealed that chrysosplenetin (CHR) significantly augmented the rat plasma level and anti-malarial efficacy of artemisinin (ART), partially due to the uncompetitive inhibition effect of CHR on rat CYP3A. But the impact of CHR on P-gp is still unknown.

**Objective:** The present study investigates whether CHR interferes with P-gp-mediated efflux of ART and elucidates the underlying mechanism.

**Materials and methods:** P-gp-over-expressing Caco-2 cells were treated with ART (10 μM) or ART-CHR (1:2, 10:20 μM) in ART bidirectional transport experiment. ART concentration was determined by UHPLC-MS/MS method. Healthy male ICR mice were randomly divided into nine groups (*n* = 6) including negative control (0.5% CMC-Na solution, 13 mL/kg), ART alone (40 mg/kg), verapamil (positive control, 40 mg/kg), ART-verapamil (1:1, 40:40 mg/kg), CHR alone (80 mg/kg), ART-CHR (1:0.1, 40:4 mg/kg), ART-CHR (1:1, 40:40 mg/kg), ART-CHR (1:2, 40:80 mg/kg) and ART-CHR (1:4, 40:160 mg/kg). The drugs were administrated intragastrically for seven consecutive days. MDR1 and P-gp expression levels in mice small intestine were examined by performing RT-PCR and western blot analysis. ABC coupling ATPase activity was also determined.

**Results:** After combined with CHR (1:2), P_app_ (AP-BL) and P_app_ (BL-AP) of ART changed to 4.29 × 10 ^−^ ^8^ (increased 1.79-fold) and 2.85 × 10 ^−^ ^8 ^cm/s (decreased 1.24-fold) from 2.40 × 10 ^−^ ^8^ and 3.54 × 10 ^−^ ^8 ^cm/s, respectively. Efflux ratio (P_BA_/P_AB_) declined 2.21-fold (*p* < 0.01) versus to ART alone. ART significantly up-regulated both MDR1 mRNA and P-gp levels compared with vehicle, while CHR in combination ratio of 0:1, 0.1:1, 1:1, 2:1 and 4:1 with ART, reversed them to normal levels as well as negative control (*p* < 0.05). The ATPase activities in ART-CHR 1:4 and CHR alone groups achieved a slight increase (*p* < 0.05) when compared with ART alone.

**Discussion and conclusion:** These results confirm that CHR inhibited P-gp activity and reverse the up-regulated P-gp and MDR1 levels induced by ART. It suggested that CHR potentially can be used as a P-gp reversal agent to obstruct ART multidrug resistance.

## Introduction

**Figure 1.**
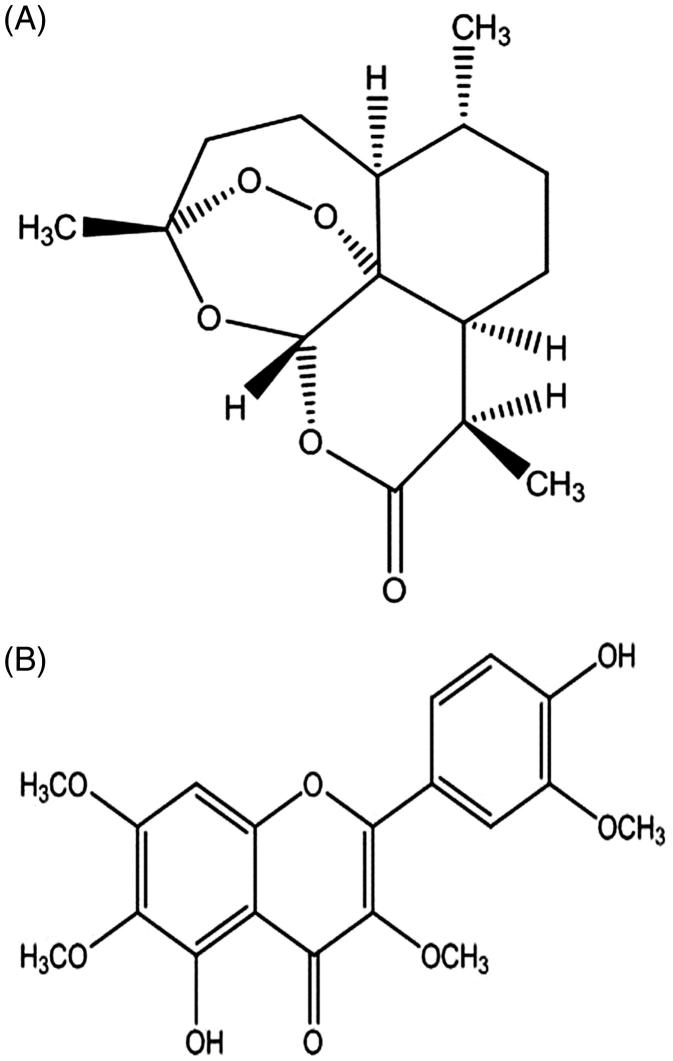
Structures of ART (A) and chrysosplenetin (B).

To date, artemisinin (ART, Figure 1(A)) antimalarial drugs are still of the utmost importance in the worldwide combination therapy of resistant *Plasmodium falciparum* (Tripathi et al. 2013). Unfortunately, ART resistance defined as a delayed clearance of parasites after clinical therapy has been reported (Meshnick et al. 1996; White 2004). The mechanism of ART resistance, however, is nearly unknown and probably modulated by multiple mechanisms, which mainly involved multidrug resistance proteins such as several members of the ATP-binding cassette (ABC) transporter super-family (Burk et al. 2005; Alcantara et al. 2013). Among them, P-gp (MDR1) is the most extensively studied efflux transporter responsible for limiting the intestinal absorption of a diverse range of xenobiotics. P-gp usually shares the identical substrates with human CYP3A4/rat CYP3A (Pal et al. 2011; Nabekura et al. 2015; Wang et al. 2015). This causes the low bioavailability and blood concentration for terminal drugs (Meng et al. 2014). Burk et al. (2005) suggested that ART induced the expression of CYP2B6, CYP3A4 and MDR1 through activating human PXR as well as human and mouse CAR as a ligand of both two nuclear receptors.

Many polymethoxylated flavonoids are able to modulate the activity of drug-metabolizing enzymes and ABC transporters, which raises the potential for alterations in the pharmacokinetics of substrate drugs (Li et al. 2010; Wesolowska 2011; Yuan et al. 2012). Chrysosplenetin (CHR, Figure 1(B)) is one of the polymethoxylated flavonoids in *Artemisia annua* L. (Compositae) and other several Chinese herbs (Numonov et al. 2015). In our previous study (Chen et al. 2014), CHR was observed to be abundant in the acetone layer, among which of the five parts of waste materials (petroleum ether layer, petroleum ether–acetate ethyl layer, ethanol layer, acetone layer and the residues after extraction) produced during ART industrial purification from leaves of *A. annua*. A patent for the purification of CHR from the acetone layer was granted in 2014 (Chen et al. 2014). Furthermore, CHR significantly increased rat plasma concentration of ART and its anti-malarial efficacy, partially due to the inhibition effect of CHR on rat CYP3A in an uncompetitive manner (Wei et al. 2015).

In the present research, we further confirmed the inhibition of CHR on P-gp-mediated efflux of ART in P-gp-over-expressing Caco-2 cells and the reversal of CHR on the up-regulated MDR1 and P-gp levels induced by ART in small intestine of mice. Our study provides a better understanding of CHR function as a potent small molecular inhibitor on P-gp-mediated ART multi-drug resistance.

## Materials and methods

### Animals

Healthy male ICR mice (18–22 g of body weight) were purchased from SPF Animal Centre of Ningxia Medical University (Ningxia, China). The permission number was SCXK 2010-0002. All animals were housed in polycarbonate cages and acclimated in an environmentally controlled room (23 ± 2 °C, with adequate ventilation and a 12 h light/dark cycle) prior to use. All animals were provided with standard laboratory food and water before and during the experiments. The experimental protocol was approved by the University Ethics Committee. All procedures involving animals were in accordance with the Regulations of the Experimental Animal Administration, State Committee of Science and Technology. Animals were randomly divided into nine groups (*n* = 6 for each group) including negative control (0.5% CMC-Na solution, 13 mL/kg), ART alone (40 mg/kg), verapamil (positive control, 40 mg/kg), ART-verapamil (1:1, 40:40 mg/kg), CHR alone (80 mg/kg), ART-CHR (1:0.1, 40:4 mg/kg), ART-CHR (1:1, 40:40 mg/kg), ART-CHR (1:2, 40:80 mg/kg) and ART-CHR (1:4, 40:160 mg/kg). The drugs were administrated intragastrically for seven consecutive days.

### Materials and instruments

Verapamil hydrochloride tablets (No.140101) were purchased from Guangdong Hua-nan Pharmaceutical Group Co., Ltd (Guangdong China). ART (white crystal, purity >99.0%, Chongqing, China) was purchased from Chongqing Huali Konggu Co., Ltd. (Beijing, China). CHR (yellow crystal, purity >98.0%) was purified in our lab from an acetone layer of ART industrial waste materials using multiple column chromatography methods with yield around 1% according to our patent (Chen et al. 2014). The industrial wastes were kindly provided by Chongqing Huali Konggu Co., Ltd and the voucher specimen has been deposited with College of Pharmacy, Ningxia Medical University, for further references.

### Bidirectional transport experiments in P-gp-over-expressing Caco-2 cell monolayers

Cells were seeded in the transwell polycarbonate inserts at a density of 10^6^ cells per well and were grown in a culture medium consisting of Dulbecco’s modified eagle’s medium supplemented with 10% foetal bovine serum, 1% non-essential amino acids, 1% l-glutamine, 100 U/mL penicillin-G and 100 μg/mL streptomycin. The culture medium was replaced every alternate day and the cells were maintained at 37 °C, 95% relative humidity and 5% CO_2_. Permeability studies were conducted with the monolayers cultured for 19–21 d. Monolayers with transepithelial electrical resistance (TEER) values higher than 420 Ω/cm^2^ were used for the experiments. Before the experiments, cell monolayers were washed twice with warm HBSS and equilibrated with HBSS for 30 min at 37 °C. HBSS containing ART (10 μM) or ART-CHR (1:2, 10:20 μM) was added to either the apical or basolateral chamber (donor chamber), and blank HBSS was added to the opposite chamber (receiver chamber). The total volume is 2.5 mL in apical chamber and 2.5 mL in the basolateral chamber. A 200 μL aliquot of samples was separately taken from the donor and receiver chambers each 1 h until 6 h and the same volume (200 μL) of blank HBSS was immediately added, respectively. Transport experiments were conducted in an incubator maintained at 37 °C and shaken with a speed of 50 rpm. The samples were dried in N_2_ and the residues were resolved by 50% methanol under a 30 s vortex. About 20 μL of blank HBSS and 200 μL of IS (daidzein, 0.5 μM) were added into 20 μL of samples. After a 30 s vortex, the mixture was centrifuged at 10,000 rpm and 4 °C for 10 min. The concentration of ART was determined by UHPLC-MS/MS.

P_app_ values across the cell monolayers were calculated according to the equation:

$$P_{\mathrm{app}}\left(\mathrm{cm}/s\right)\;=\;\frac{\mathrm{dQ}}{\mathrm{dt}}\;\times \;\frac{1}{A\times C_{0}},$$

where *dQ/dt* represents the rate of drug transport. *A* is the surface area of the cell monolayer (4.2 cm^2^). *C*_0_ is the initial concentration of ART in the donor chamber. Efflux ratio was calculated by dividing P_app_ (BL-AP) by P_app_ (AP-BL). P_app_ (AP-BL) represents the transport of ART from the apical to basal side and P_app_ (BL-AP) represents that from the basal to apical side.

### Determination of ART using UHPLC-MS/MS method

The UHPLC conditions were as follows: Waters Acquity^TM^ with diode array detector (DAD); column, Acquity UHPLC BEH C_18_ column (50 mm ×2.1 mm I.D., 1.7 μm, Waters, Milford, MA); mobile phase A (0.1% formic acid in water); mobile phase B (acetonitrile); gradient, 0–0.5 min, 95% B; 0.5–2.0 min, 95–40% B; 2.0–4.0 min, 40–90% B; 4–4.5 min, 90–5% B; 4.5–5 min, 95% B; column temperature, 45 °C; sample temperature, 20 °C; and injection volume, 10 μL (Sun et al. 2015).

**Figure 2.**
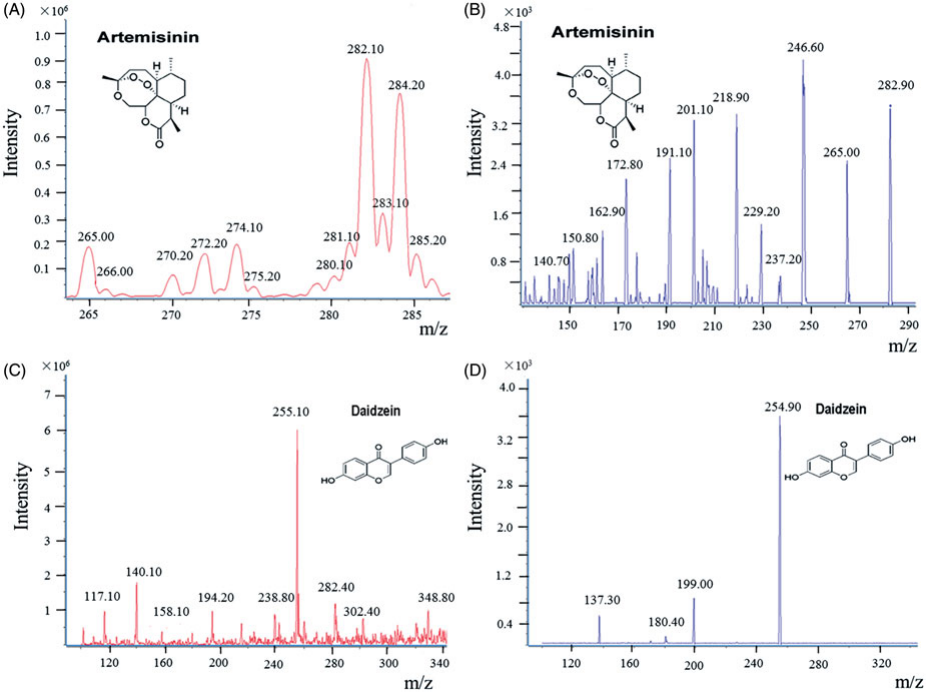
Collision-induced dissociation mass spectra for ART (A: MS^1^ and B: MS^2^) and daidzein (C: MS^1^ and D: MS^2^).

The MS analysis was performed on an API 5500 Qtrap triple quadrupole mass spectrometer (Applied Biosystem/MDS SCIEX, Foster City, CA) equipped with TurboIon Spray^TM^ source. The compounds were determined by using multiple reaction monitoring (MRM) scan type in positive mode. The instrument-dependent parameters for mass spectrum were set as follows: ion-spray voltage, 5.5 kV; ion source temperature, 400 °C; nebulizer gas (gas 1), nitrogen, 40 psi; turbo gas (gas 2), nitrogen 20 psi; curtain gas, nitrogen 20 psi. The collision energy was 20 eV for ART and 32 eV for IS. Base on the full-scan mass spectra of each analyte, the MRM transition *m/z* 2 8 3 → 151 (Figure 2(A) and (B)) for ART was selected for quantitative analysis of ART and *m/z* 2 2 5 → 199 for daidzein (Figure 2(C) and (D)).

### RNA extraction and quantitative polymerase chain reaction (qPCR)

The mice were euthanized by cervical vertebra dislocation. Small intestines were harvested and cleaned using normal saline at least three times. Total RNA was extracted from the mouse small intestine using E.Z.N.A.^TM^ Total RNA Kit (OMEGA Bio-Tek, Norcross, GA), in accordance with the instructions of the manufacturer. RNA concentrations were measured with a microplate spectrophotometer (Bio-RAD, Hercules, CA) at 260 nm. RNA quality was evaluated using electrophoresis in 1% agarose gels. Total RNA (3 μg) was reverse transcribed into first-strand complementary DNA (cDNA) using Thermo Scientific RevertAid First Strand cDNA Synthesis Kit (Thermo Scientific, Waltham, MA). Each cDNA sample (1 μL) was amplified with 12 μL Thermo Scientific Maxima SYBR Green qPCR Master Mix (2×). ROX Solution provided (Thermo Scientific, Waltham, MA) and 1 μmol of each primer. Amplification was performed in a Real Time PCR IQ5 System (Applied Biosystems, Foster City, CA) with the following parameters: denaturation at 94 °C for 2 min followed by 35 cycles of denaturation at 94 °C for 30 s, annealing at 61 °C for 30 s and extension at 72 °C for 45 s. The sequences of the oligonucleotide primers used for this study were 5′-GGG CAC AAA CCA GAC AAC AT-3′ (sense) and 5′-TCC GCT CTT CAC CTT CAG AT-3′ (antisense) for MDR1 (product size, 117 bp from Sangon Biotech (Shanghai) Co., Ltd., Shanghai, Japan); and 5′-GGT GAA GGT CGG TGT GAA CG-3′ (sense) and 5′-CTC GCT CCT GGA AGA TGG TG-3′ (antisense) for GAPDH (product size, 233 bp, from Invitrogen Biotechnology Co., Ltd, Carlsbad, CA). The relative expression levels of MDR1 in each sample (normalized to that of GAPDH) were determined using 2^-ΔΔCt^ method (Inami et al. 2015; Zhang et al. 2015; Hao et al. 2016). All qPCR experiments were repeated three times.

### Determination of the expression levels of P-gp by western blot analysis

Membrane proteins were harvested by KenGEN Membrane and Plasma Protein Purification Kit (KenGEN BioTECH, Nanjing, China), and the protein concentrations were determined using KeyGEN BCA Protein Quantitation Assay kit (KenGEN BioTECH, Nanjing, China). An equal quantity of protein (48 μg) from membrane protein was resolved using 7.5% SDS-PAGE gel and subsequently transferred onto nitrocellulose membranes (Bio-Trace, Auckland, New Zealand). After blocking the membrane with 5% non-fat milk in Tris-buffered saline (Biotopped) at room temperature for 1 h, the membrane was incubated at 4 °C for 12 h with rabbit monoclonal primary antibodies against MDR1 (1:1250; ab170904) and Anti-Sodium Potassium ATPase antibody-Plasma Membrane Loading Control (1:20,000; ab76020). Antibodies were both purchased from Abcam (Cambridge, United Kingdom). The membranes were incubated with horseradish peroxidase-conjugated AffiniPure Goat Anti-Rabbit IgG (ZSGB-BIO, Beijing, China) for 1 h and signals were observed using SuperSignal West Pico (Thermo Scientific, Waltham, MA). Western blotting bands intensity was quantified by densitometric analysis using Image J version 2 × (NIH Image Software, Bethesda, MA).

### ABC transporter-related ATPase activity assay

The activity of ABC transporter coupling ATPase was measured by monitoring the release of inorganic phosphate (Pi) from adenosine triphosphate (ATP) by ABC transporter membranes in the presence or absence of vanadate (Xia et al. 2007). Mice small intestine was homogenized with glass homogenizer (50 up/down strokes) to prepare membrane proteins using KenGEN Membrane and Plasma Protein Purification Kit (KenGEN BioTECH, Nanjing, China). Protein concentration was determined by using BCA Protein Quantitation Assay Kit (KenGEN BioTHCH, Nanjing, China). A 200 μL diluted membrane protein was pre-incubated in 200 μL inhibitor mix (4 mM ouabain, 4 mM EGTA, 10 mM NaN_3_, adjust to pH = 7). The ATPase reaction was measured in the presence or absence of 0.05 mM Na_3_VO_4_ (vanadate) using Microscale Total ATPase Assay Kit (Nanjing Jiancheng BIoeng Inst., Nanjing, China) according to the instructions of the manufacturer. Briefly, 0.1 mL of the sample was pre-incubated in 0.42 mL assay buffer for 10 min at 37 °C. ATPase hydrolysis was accurately processed for 10 min by adding 0.1 mL of ATP stock solution, and then 100 μL of Reagent Four was immediately added to the mixture to end reaction. Mixture was centrifuged at 4000 rpm for 10 min and 0.3 mL of supernatant was transferred to 1 mL of chromogenic agent solution, incubated for 2 min at ambient temperature and the reaction was terminated with 1 mL of Reagent Six. The concentration of Pi was measured by the absorbance (*A*) at 636 nm wavelength and acquired by fitting in standard curve generated with series of Pi standard solution of varied concentrations.

### Statistical analysis

All data were analyzed using the SPSS 18.0 software (IBM, Armonk, NY). Data were indicated as mean ± SD. *T*-test obtained by one-way analysis of variance (ANOVA). *p* < 0.05 and *p* < 0.01 were considered to be statistically different.

## Results

### UHPLC-MS/MS analysis

**Figure 3.**
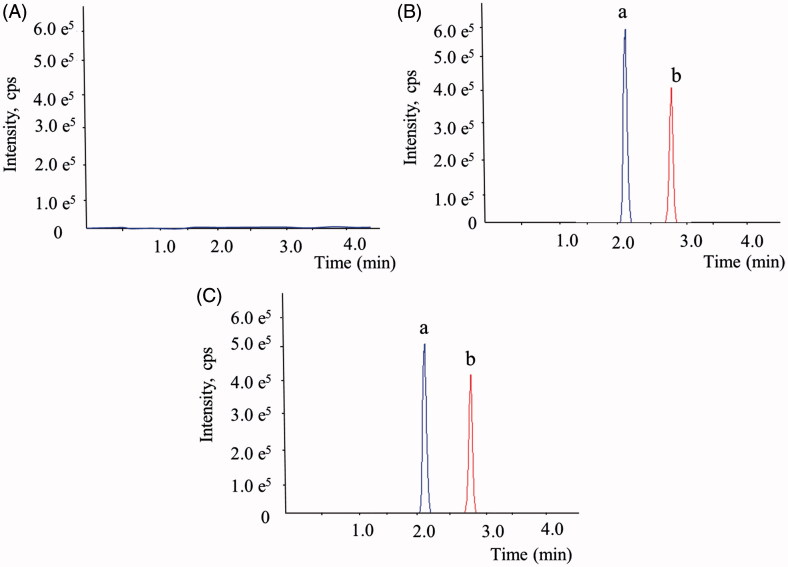
Representative full-scan chromatograms of (A) blank HBSS, (B) blank HBSS spiked with ART (a) and daidzein (b, IS) and (C) a study sample containing ART (a) and daidzein (b, IS) after incubation for 1 h.

Under optimized UHPLC conditions, ART and daidzein were eluted within 3.5 min as shown in Figure 3(B) and (C). In Figure 3(A), blank HBSS showed no interfering peaks at the retention times of each analyte. The calibration curve of ART was linear in the concentration range of 3.91–2000.00 nM (*y* = 0.000412× + 0.00354, *r* = 0.9932, *w* = 1/*x*^2^). All coefficients of variation at each concentration are below 10%.

### Bidirectional transport assay in P-gp-over-expressing Caco-2 cell monolayers

Table 1 shows the permeability of ART across P-gp-over-expressing Caco-2 cell monolayers. P_app_ (AP-BL) values of ART from apical-to-basal side and P_app_ (BL-AP) from basal-to-apical side were 2.40 × 10 ^−^ ^8^ and 3.54 × 10 ^−^ ^8 ^cm/s at 10 μM, respectively. For the ART-CHR combination group, P_app_ (AP-BL) and P_app_ (BL-AP) of ART became 4.29 × 10 ^−^ ^8^ (increased 1.79-fold) and 2.85 × 10 ^−^ ^8 ^cm/s (decreased 1.24-fold), respectively. Efflux ratio (P_BA_/P_AB_) in the ART-CHR combination group achieved 2.21-fold decrease (*p* < 0.01) versus to ART alone. Thus ART has poor passive diffusion ability (Papp <10 ^−^ ^7^ and efflux ratio <2). Moreover, P-gp involved in ART efflux could be inhibited by CHR.

**Table 1.** Permeability of ART across P-gp-over-expressing Caco-2 cell monolayers in the absence and the presence of CHR (mean ± SD, *n* = 3).

	Papp (× 10^−8^cm/s)		
Drugs	AP-BL	BL-AP	P_BA_/P_AB_(efflux ratio)
ART (10 μM)	2.40 ± 0.21	3.54 ± 0.27^##^	1.48 ± 0.07
CHR-ART (2:1, 20:20 μM)	4.29 ± 0.16**	2.85 ± 0.04^##,^*	0.67 ± 0.03**

*p < 0.05 and **p < 0.01 versus ART; ^##^*p* < 0.01 versus P_app_ (AP-BL).

### MDR1 expression determined by RT-QPCR

**Figure 4.**
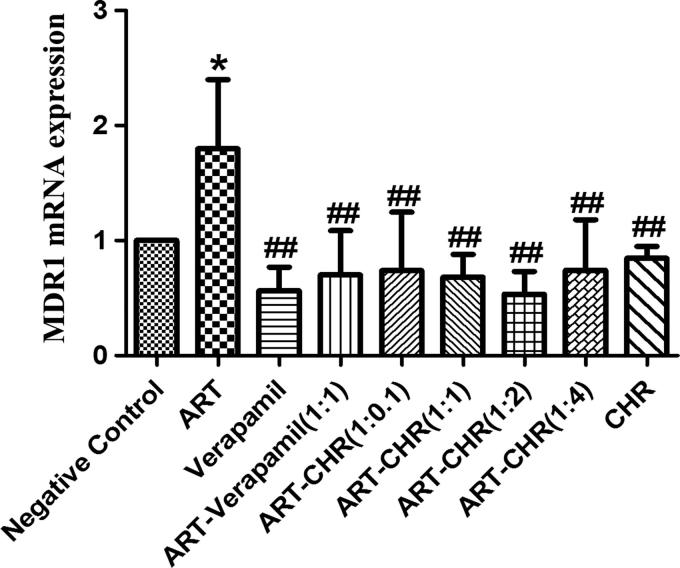
The expression of MDR1 mRNA in small intestine. MDR1 mRNA levels were determined by real-time qPCR after seven days oral xenobiotic pre-exposure in ICR mice. 0.5% CMC-Na was used as negative control and verapamil as positive control. All values were expressed as the mean ± SD (*n* = 6) for each group. *p < 0.05 compared with negative control. #*p* < 0.05, and ##*p* < 0.01 versus ART alone.

As represented in Figure 4, ART alone significantly increased the level of MDR1 mRNA compared with the negative control (*p* < 0.05). No significant difference was observed among negative and positive control or combined groups. When compared with ART alone, however, the MDR1 mRNA expressions in positive control (verapamil, ART-verapamil) and ART-CHR combined groups (1:0, 1:1, 1:2, 1:4, 0:1) were reversed to normal levels as well as negative control (*p* < 0.01). CHR, therefore, could reverse the MDR1 up-regulated expression by ART. The lowest MDR1 mRNA level was obtained when the combined ratio of ART and CHR was 1:2. It suggested that binding sites of P-gp might be saturable.

### Determination of P-gp levels by western blot analysis

**Figure 5.**
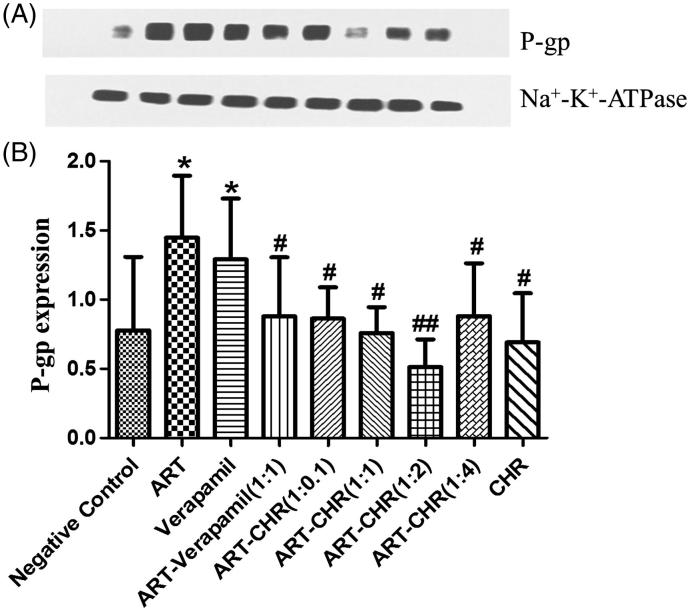
Impact of CHR on P-gp expression. (A) Western blot bands. (B) Quantification of P-gp assessed by Western blotting analysis was normalized to the expression level of Na^+^–K^+^–ATPase antibody. All values were expressed as the mean ± SD (*n* = 6) for each group. *p < 0.05 versus negative control. #*p* < 0.05 and ##*p* < 0.01 versus ART alone.

As shown in Figure 5(A) and (B), P-gp expression was up-regulated by ART alone (*p* < 0.05) and verapamil (*p* < 0.05). No significant difference was found among negative control and ART-verapamil or ART-CHR combined groups or CHR alone (*p* > 0.05). Compared with ART alone, however, P-gp expression levels in ART-verapamil (1:1), ART-CHR (1:0.1, 1:1, 1:2, 1:4) and CHR alone significantly decreased (*p* < 0.05 and *p* < 0.01). In the presence and absence of CHR or verapamil, P-gp expression showed a significant difference. The results suggest that verapamil and CHR are capable of reversing ART-induced P-gp up-regulation.

### ABC transporter-related ATPase activity assay

**Figure 6.**
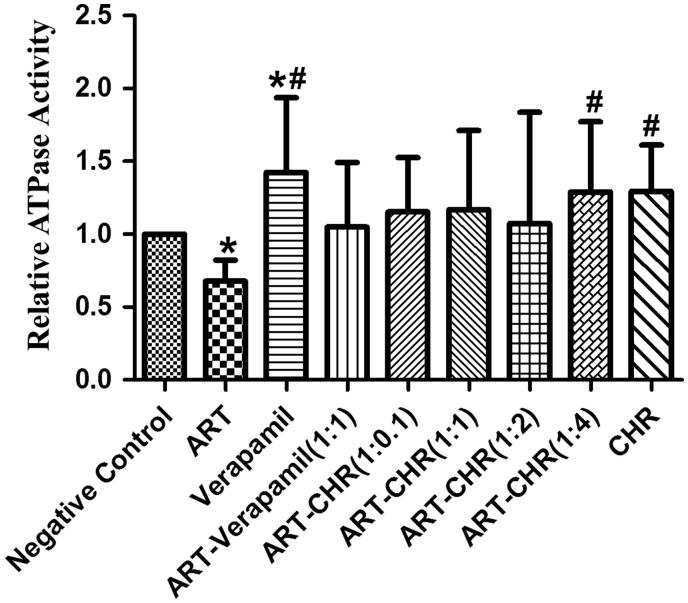
ABC transporter coupling ATPase activity in the mice small intestine. The impact of CHR in the absence and presence of ART on ATPase activity was investigated. *p < 0.05 versus negative control. ##*p* < 0.05 versus ART alone.

Since ABC transporters are ATP-driven active transporters, ATPase activity is required to fuel the transportation activity. To test whether CHR influence the ABC transporter-related ATPase activity, microscale total ATPase assay kit was used to measure the ATPase activity. As shown in Figure 6, verapamil alone stimulates the ATPase activity (*p* < 0.05) while ART alone remarkably decreased the ATPase activity versus the vehicle (*p* < 0.05). When compared with ART alone, the ATPase activities determined in ART-CHR 1:4 and CHR alone groups achieved a slight increase (*p* < 0.05).

## Discussion

CHR belongs to the polymethoxylated flavonoids together with ART in the leaves and flowers of *A. annua*. The acetone layer of the industrial waste of ART is enriched with CHR. However, these industrial wastes are always discarded because few researchers focus on it. More recently, accumulating evidence suggested that many flavonoids have the ability to inhibit CYP3A4 and P-gp activity (Sandor et al. 1998; Middleton et al. 2000; Daddam et al. 2014). Here, we have demonstrated that CHR exerts an inhibition not only on the rat CYP3A-mediated metabolism of ART as previously shown but also on its P-gp-mediated efflux through reversing the up-regulated P-gp and MDR1 expression levels induced by ART.

Interestingly, the lowest P-gp and MDR1 levels were both observed when the combination ratio between ART and CHR was 1:2. It is in accordance with our previous work (Wei et al. 2015). It was reported that the AUC_0–t_, *C*_max_, and *t*_1/2_ of ART increased significantly as well as declined CLz after 3-d oral doses of ART in the presence of CHR (1:2) when compared with ART alone. Also, parasitaemia (%) remarkably attenuated 1.59-fold with 1.63-fold augmented inhibition (%) only when the ratio between ART and CHR reached 1:2. The results provided a direct proof that CHR in combination ratio of 1:2 with ART has a strong reversal effect on P-gp-mediated efflux of ART by down-regulating P-gp and MDR1 mRNA levels.

Verapamil, a classic substrate and inhibitor of P-gp, is a calcium channel blocker. It was reported that verapamil can increase the accumulation of Rh-123 in Caco-2 cells or MDCK-MDR1 (Chieli et al. 2012; Hu et al. 2016; Miao et al. 2016); therefore, it was always used as a reference compound to develop other P-gp substrates. Although verapamil inhibits P-gp function, several reports revealed that verapamil increased the P-gp level when it was applied alone (Collett et al. 2004; Grybauskas et al. 2015; Mohseni et al. 2016). Our results completely conform to the literatures. But recently, Miao et al. (2016) reported that verapamil down-regulated P-gp expression in Caco-2 cells. Therefore, the effect of verapamil on P-gp and MDR1 mRNA expression is still unclear.

P-gp is a member of the energy-dependent efflux pump associated with the excretion of many P-gp substrates and non-substrates, so ATPase activity plays an important role in the mechanisms of P-gp inhibition (Suri et al. 2008). As shown in Figure 6, ART inhibited the ATPase activity whereas verapamil significantly stimulated it. Our data are in consistent with the results reported previously (Burk et al. 2005). CHR alone and ART-CHR in ratio of 1:4 had a slight stimulation on ATPase activity. Hence CHR as a P-gp inhibitor merely has a weak stimulating effect on ATPase activity.

In conclusion, CHR inhibits P-gp-mediated ART efflux through reversing MDR1 mRNA and P-gp up-regulated expression induced by ART and has very weak stimulation on ATPase activity. CHR, therefore, can be used as a promising P-gp small molecular inhibitor in the future. More importantly, the industrial waste of ART could be fully used to produce CHR: in fact it might be useful in reducing the cost of ART production.
